# Overexpression of RhoV Promotes the Progression and EGFR-TKI Resistance of Lung Adenocarcinoma

**DOI:** 10.3389/fonc.2021.619013

**Published:** 2021-03-09

**Authors:** Hongjin Chen, Ruixue Xia, Long Jiang, Yong Zhou, Haojun Xu, Weiwei Peng, Chengyun Yao, Guoren Zhou, Yijie Zhang, Hongping Xia, Yongsheng Wang

**Affiliations:** ^1^ Department of Pathology, School of Basic Medical Sciences & Sir Run Run Hospital & Key Laboratory of Antibody Technique of National Health Commission, Nanjing Medical University, Nanjing, China; ^2^ Department of Respiratory and Critical Care Medicine, Henan University Huaihe Hospital, Kaifeng, China; ^3^ Shanghai Lung Cancer Center, Shanghai Chest Hospital, Shanghai Jiao Tong University, Shanghai, China; ^4^ Nanjing Drum Tower Hospital Affiliated to Medical School of Nanjing University, Nanjing, China; ^5^ Jiangsu Cancer Hospital & The Affiliated Cancer Hospital of Nanjing Medical University & Jiangsu Institute of Cancer Research, Nanjing, China

**Keywords:** RhoV, lung adenocarcinoma, gefitinib, AKT, ERK

## Abstract

**Background:**

The Rho GTPase family with ~20 member genes play central roles in a wide variety of cellular processes and tumor cell migration and metastasis. Different Rho GTPase may play different roles in the progression of lung adenocarcinoma.

**Methods:**

We comprehensively examined the expression of all Rho GTPase family member genes in a panel of lung adenocarcinoma patient’s tumors and matched normal tissues. We next investigated the critical role of RhoV in different lung adenocarcinoma cells and animal models.

**Results:**

RhoV was identified as one of the most significantly overexpressed Rho GTPases in lung adenocarcinoma and associated with patients’ survival. Silencing RhoV expression inhibits proliferation, migration and invasion, and tumorigenicity capacities of lung adenocarcinoma cells. Moreover, knockdown RhoV promoted the sensitivity of EGFR-TKI in the gefitinib resistant PC9 cells (PC9-GR) and aggravated gefitinib-induced lung cancer cell apoptosis both in PC9 and PC9-GR cells. Our data also indicated that RhoV induced progression and EGFR-TKI resistance of lung adenocarcinoma may be related to the activation of the AKT/ERK pathway.

**Conclusion:**

Overexpression of RhoV in lung adenocarcinoma promotes the progression and EGFR-TKI resistance, suggesting RhoV is a promising prognosis and therapeutic target of lung adenocarcinoma.

## Background

Lung cancer, a major health risk worldwide, is the most common malignancy and continues to be the leading cause of cancer-associated deaths (1), and the incidence in China is also increasing in recent years (2). It can be divided into non-small cell lung cancer (NSCLC) and small cell lung cancer (SCLC) by histological type in clinical. NSCLC represents approximately 85% of all lung cancer patients and the overall 5-year survival rate of 10–15%. According to the histological types, NSCLC is divided into lung adenocarcinoma (LUAD, 40–50%) and lung squamous cell carcinoma (LUSC, 30%) (3). Currently, there have been great strides and treatment with some effective means, like surgical treatment, radiotherapy, and chemotherapy, in advancing modalities for NSCLC (4, 5). However, the early diagnosis rate of LUAD is low for the reason that lack of typical early clinical symptoms and signs, meanwhile, unclearly mechanism is a significant challenge for this cancer treatment.

The Rho GTPase family is a member of the Ras superfamily. It is a type of protein that binds to GTP and can exert a variety of biological effects through its downstream effector proteins. It can play a “molecular switch” in the process of signal transmission in eukaryotic cells. Some Rho family members, like RhoA and RhoC, have been testified that are strongly associated with the onset, development and migration of lung cancer (6). RhoV, also named Chp, has been shown to promote cell differentiation and as an essential regulator of neural crest induction (7, 8), which indicated that RhoV is involved in cell development. Meanwhile, RhoV also was investigated that exhibits high expression in many lung cancer cell lines (9). However, it is rarely known about the functional role of RhoV in LUAD until now, and the mechanism still had not been reported.

Epidermal growth factor receptor (EGFR) has been confirmed to be of great importance in cell differentiation, cellular immune response, proliferation, and apoptosis. Moreover, growing papers testified that EGFR is high-expression in lung cancer patients, induces excessive activation to many signaling pathways, like AKT/mTOR and MAPK/ERK (10). Gefitinib is the most efficient treatment for block EGFR activation in clinic cases (11). However, gefitinib resistance (GR) is inevitably exhibited in most patients. Recent evidence implicates GTP-energy abnormality as playing roles in cancer drug resistance, whereas this in LUAD remains poorly understood. Some papers showed that RhoV could influence Pak kinases activation, like Pak1 and Pak6, which are the biomarkers of NSCLC, to regulate cell development (12, 13). Therefore, we hypothesize that RhoV could participate in the LUAD resistance of gefitinib.

In this study, we found that RhoV was identified as one of the most significantly overexpressed Rho GTPases in lung adenocarcinoma. Meanwhile, we investigated the critical roles of RhoV on the growth and metastasis of different lung adenocarcinoma cells. Besides, we explored that knockdown RhoV increased the sensitivity of gefitinib in PC9 cells as well as the underlying mechanism mainly focuses on the phosphorylation of AKT/ERK. Our study indicated that RhoV could be a tumor activator and a potential target for the treatment of human lung cancer.

## Materials and Methods

### Bioinformatic Analysis

Gene expression profiling interactive analysis (GEPIA, http://gepia.cancer-pku.cn/) is a free open-access website for the analysis of cancer genomics data. To analyze the expression of RhoV and related-survival curve in the TCGA LUAD and LUSC samples were searched in GEPIA. The GSE18842 dataset was downloaded on the GEO websites and analyzed by Partek^®^ Genomics Suite^®^ (Partek SG Pte. Ltd. Singapore).

### Cell Lines

Beas-2B, H1975, Calu1, A549, H1299, PC9, and PC9-GR (Gefitinib resistant) cell lines were obtained from our lab. The PC9 [EGFR exon 19 deletion (delE746-A750)] and its gefitinib-resistant cell line PC9GR were used as our previous description (14). All cell lines were cultured in DMEM medium, with 10% fetal bovine serum (FBS; Gibco; Thermo Fisher Scientific, Inc., USA) and 1% penicillin/streptomycin (Gibco; Thermo Fisher Scientific, Inc., USA), under 37°C in a humidified atmosphere containing 5% CO_2_.

### Transient Transfection of esiRNA and Plasmid Transfection

RhoV esiRNA (No. EHU054401) was purchased from Merck (Darmstadt, Germany). Lipofectamine RNAiMAX (Invitrogen, Carlsbad, CA) was used to transfected esiRNA according to the manufacturer’s instructions. Scramble siRNA was taken as a negative control.

For lentivirus infection experiments, the TransIT-293 transfection reagent (Mirus, Madison, USA) was utilized to transfect shRhoV (5’-ATGTCTTCCTGGCGTGCTTCA-3’), shScramble, and packaging plasmid into 293T cells. After the transfection 48 h, lentiviruses were used to infect cells at 24 h. To screening stably infected cells, they were subjected to the treatment of puromycin (3 μg/ml) for 3 days. Surviving cells were been stable transfection cells for further experiments.

For overexpression (OE) assay, the OE plasmid of RhoV (No. RC211121) was purchased from Gene-Ethics (Asia) Private Limited (Singapore). The Lipofectamine 2000 (No. 11668019, Thermo Fisher Scientific, USA) was used to transfect OE-RhoV plasmid into cells and the overexpression efficiency was confirmed by western blot assay after transfected 48 h.

### Real-Time Quantitative PCR

RNAzol^®^ reagent (Sigma-Aldrich, USA) was used to extract total RNA. Then using 5X All-In-One RT MasterMix or BrightGreen Express 2X qPCR MasterMix-Low ROX (Abm, Canada), total RNA was reversed into cDNA and real-time quantitative PCR was performed by the manufacturer’s instructions. The primers of human genes, including RhoV and β-actin, were synthesized from Integrated DNA Technologies (IDT, Singapore). The primer sequences used are shown as follows: human RhoV sense primer: 5′- CCTCATCGTCAGCTACACCTG -3′, human RhoV antisense primer: 5′- GAACGAAGTCGGTCAAAATCCT -3′; human β-actin sense primer: 5′- CCTGGCACCCAGCACAAT -3′, human β-actin antisense primer: 5′- GCCGATCCACACGGAGTACT -3′. After the amount of each gene was determined, it was normalized to the amount of β-actin.

### Western Blot

RhoV antibody (No. 26620-1-AP) was purchased from ProteinTech (Wuhan, China). Anti-Ki67 (No. 9449), anti-p-AKT Ser473 (No. 4060), anti-Vimentin (No.5741), and anti-Tublin (No. 2148) were purchased from Cell Signaling Technology (Danvers, MA, USA). Anti-p-EGFR Y1068 (No. AP0301), anti-EGFR (No. A11351), and anti-p-ERK (AP0974) were obtained from Abclonal (Wuhan, China). Anti-β-Catinentin (No. 06-734) was purchased from Upstate Biotechnology (Fisher Scientific, USA). The lysate was prepared and protein concentration was ascertained by BCA kit (Sigma-Aldrich, USA). Proteins were separated by 10 or 12% sodium dodecyl sulfate-polyacrylamide gel electrophoresis and then transferred onto polyvinylidene fluoride membrane (Bio-Rad Laboratory, Hercules, CA, USA). Membranes were blocked with 5% milk in PBS with 0.05% Tween-20 (PBST), for 1.5 h at room temperature. After that, different primary antibody was incubated overnight at 4°C. Then, reaction with secondary HRP-conjugated antibody at room temperature for 1 h. The immunoreactive proteins were visualized with a chemiluminescence reagent (Bio-Rad Laboratory, Hercules, CA, USA).

### Cell Proliferation Assay

For cell viability assay, A549 and PC9 cells were seeded into 96-well plates at a density of 3 × 10^3^ per well. After 48 h, 10 μl CCK-8 reagent (Invitrogen) was added into each well and tested them after 4 h reaction at 490 nm.

For cell count assay, A549 and PC9 cells were planted at six-well plates with 10^6^ or 5×10^4^ cells per well. The cell number was counted after 72 h seeded and measured by Counter (Sigma, USA).

For the clonogenic assay, lung cancer cells (1,000/well) had been seeded into a six-well plate. After 14 days incubated, the colonies were fixed by cold ethanol for 20 min and stained with 0.5% crystal violet for 30 min at room temperature.

### Transwell Assay

A549 and PC9 cells (5 × 10^5^/well) were seeded into the serum-free medium in the upper chamber and DMEM medium containing 20% FBS in the bottom chamber. After 24 h, the invaded cells were fixed with cold methanol for 20 min. Then cells were stained with 0.5% crystal violet for 20 min. The invaded cells were counted by a microscope for three random fields (Nikon, Japan).

### Scratch Assay

Cells were seeded on a six-well plate and cultured until they reached 100% confluence. All cells were treated with serum-free medium for 24 h. Scratch assay was performed by appropriate treatment, and photographs were obtained by a microscope (Nikon) after 72 h.

### Animal Study

For tumorigenicity assay, A549 cells (5 × 10^6^/mouse), including transfected stable shRhoV and shScramble cell lines, were injected subcutaneously into NOD-SCID mice, and each group has five mice. The animal study protocol was approved by and performed following the Committee of the Use of Live Animals in Teaching and Research at the institute. Tumors size was recorded every week and calculated by the formula V = 0.5 × Length × Width^2^. After sacrificed mice, the tumors’ weight had been measured and recorded.

### Flow Cytometer Analysis

For cell apoptosis assay, PC9 and PC9-GR cells transfected esiRhoV or negative control 24 h. Cells (5 × 10^5^) were seeded in six-well cell culture plates and were treated with 3 μM gefitinib (Ger) for another 48 h. Then, PC9 and PC9-GR cells were collected and washed twice with cold PBS. The cells were resuspended in 1× binding buffer at a final concentration of 1 × 10^6^ cells/ml, transfer 500 μl of solution (5 × 10^5^ cells) to a 5 ml test tube. Then 5 μl of Allophycocyanin (APC) Annexin V (No. 550475) and 5 μl of 7-amino actinomycin D (7-AAD, No. 559925) were added to the solution and incubated at room temperature in the dark for 15 min. Then 400 μl of 1× binding buffer was added to each test tube, and the sample was analyzed by flow cytometry within 1 h. Then, we used FlowJo software to analyze all data.

### Immunohistochemistry and Immunofluorescence Analysis

The collection of clinical samples was approved by the Ethics Committee of the institute. Informed consent were obtained according to institutional guidelines. Tumors were fixed in formalin overnight, embedded in paraffin, and sectioned to 5mm. After deparaffinization and rehydration, slides were dealt with Dako REAL immunohistochemical kit (Agilent, CA, USA) according to the manufacturer’s instruction. Then, slides were incubated with specificity antibody at 4°C overnight and incubated with horseradish peroxidase-labeled secondary antibody (for immunohistochemistry) or Alexa 594-labeled secondary antibody (for immunofluorescence) for 1.5 h at room temperature.

Next, immunohistochemistry slides were visualized by DAB substrate kit (Vector Laboratories, CA, USA), and slides were counterstained with hematoxylin. While immunofluorescence slides were treated with DAPI for 15min. The images were viewed using a microscope with random three fields (Nikon).

### Statistical Analysis

All data analyses in this study were performed using Student’s t-test or website system program. The significance value of 0.05 was adopted, at which the difference is considered significant (p < 0.05).

## Results

### RhoV Is Significantly Overexpressed Rho GTPases in Lung Adenocarcinoma and Associated With Patient’s Survival

**Figure 1:**
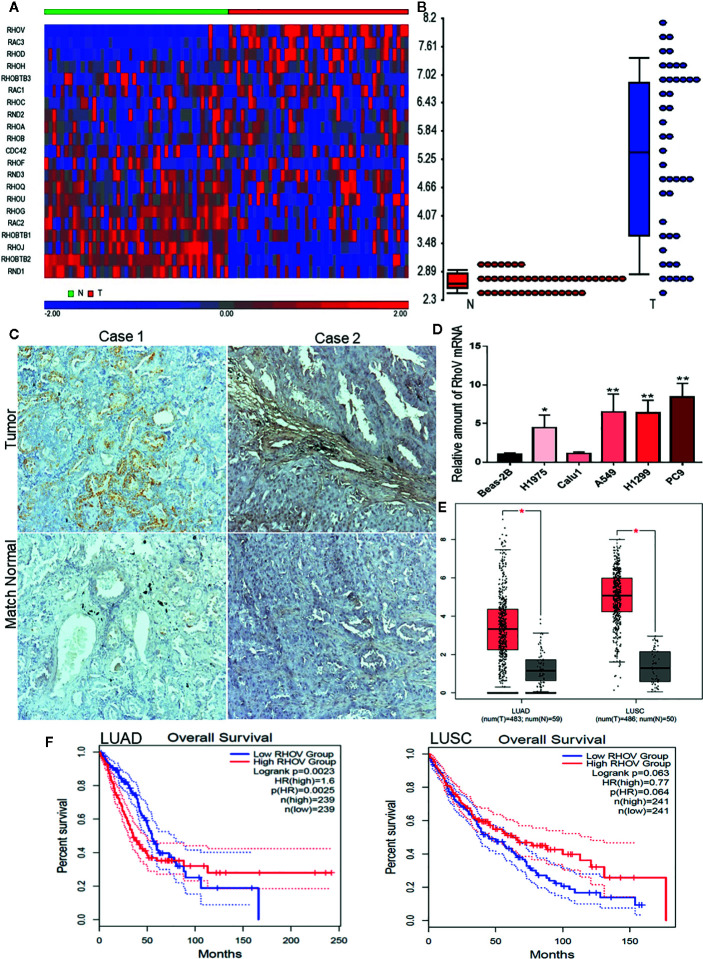
RhoV is significantly overexpressed Rho GTPases in lung adenocarcinoma and associated with patient’s survival. **(A)** The expression of all Rho GTPase family member genes based on the gene symbols was examined in a panel of 45 pairs of lung adenocarcinoma patient’s tumors and matched normal tissue samples from the GEO datasets GSE18842. **(B)** RhoV was identified as one of the most significantly overexpressed Rho GTPases in lung adenocarcinoma. **(C)** IHC staining of RhoV in the lung cancer tissues. Representative images, about match normal and tumor, were obtained at ×100 magnification (left) and ×400 magnification (right). **(D)** RT-qPCR analysis of RhoV expression in the normal human lung cell line, Beas-2B, and human LUAD cell lines. (Compared with match control, *p < 0.05, **p < 0.01). **(E)** The relative expression of RhoV in LUAD and LUSC analysis from GEPIA2 is based on the TCGA data. **(F)** Kaplan–Meier analysis of overall survival and progression-free interval time in high and low expression of RhoV groups of months.

We comprehensively examined the expression of all Rho GTPase family member genes based on the gene symbols in a panel of 45 pairs of lung adenocarcinoma patient’s tumors and matched normal tissues from the GEO datasets GSE18842 (**Figure 1A**). RhoV was identified as one of the most significantly overexpressed Rho GTPases in lung adenocarcinoma (**Figure 1B**). Meanwhile, overexpression of RhoV in LUAD has also been verified in the LUAD tissues compared with match normal tissues by IHC staining (**Figure 1C**). RhoV mRNA expression levels were also consistently increased in lots of human LUAD cell lines, such as A549, H1975, H1299, and PC9, compared with normal lung cell Beas-2B (**Figure 1D**). Next, we examined LUAD datasets from The Cancer Genome Atlas (TCGA), including 59 normal lung tissues and 481 LUAD tumor tissues. Then, 5,315, 3,901, and 1,557 of differential gene expressions (DEGs) were respectively analyzed from DESeq2, WilcoxTest, and survival analysis. Meanwhile, the three software showed that they have 525 cross DEGs (**Figure S1A**). RhoV is one of the top genes in the 525 genes. Then, the bioinformatics analysis of the volcano plot also predicted that RhoV was up-expression in LUAD (**Figure S1B**). Additionally, RhoV is significantly upregulated in LUAD and LUSC (**Figure 1E**) and correlated with poor survival of LUAD (**Figures 1F** and **S1C**). However, more RhoV expression leads to better and long survival time for LUSC patients (**Figures 1F** and **S1D**). These results initially indicated that RhoV is significantly overexpressed Rho GTPases in lung adenocarcinoma and associated with the patient’s survival.

### Knockdown RhoV Inhibited LUAD Cell Proliferation and Tumor Formation

**Figure 2:**
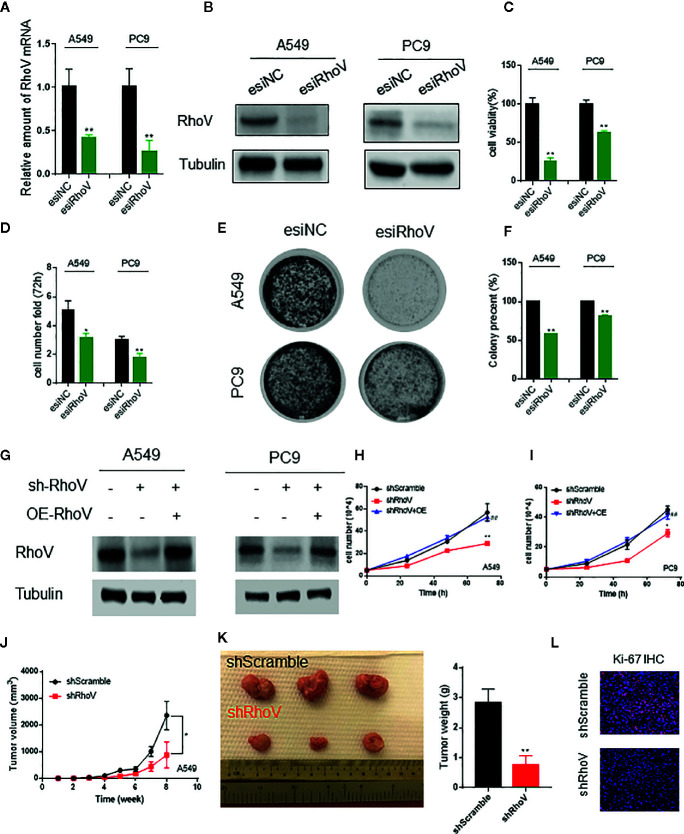
Silencing RhoV inhibited A549 and PC9 cell proliferation and tumor formation *in vivo*. **(A, B)** qRT-PCR and western blotting analysis of esiRhoV decreased the mRNA and protein expression of RhoV in A549 and PC9 cells. **(C, D)** CCK8 and cell counting analysis of silencing RhoV effect of cell viability and growth speed. **(E, F)** Colony formation was detected by crystal violet staining after the transient transfection of esiRhoV for 14 days. (Compared with match control, *p < 0.05, **p < 0.01). **(G)** Western blotting analysis of the stable knockdown and rescue effect by overexpression of RhoV in A549 and PC9 cells. **(H, I)** CCK8 analysis of the growth curve in stable knockdown RhoV and rescue by overexpression RhoV in A549 and PC9 cell lines. **(J, K)** A549 (shScramble group and shRhoV group) cells were injected subcutaneously to NOD-SCID mice at 5 × 10^6^ per mouse. Tumor volumes were measured and recorded every week (n = 5) **(J)**. At 8 weeks after injection, the mice were sacrificed, and tumors were photographed or weighed **(K)**. **(L)** IF analysis of Ki-67 expression in shScramble and shRhoV tumors. (Compared with match control, *p < 0.05, **p < 0.01; compared with shRhoV, ^##^p < 0.01).

To determine the functional role of RhoV in LUAD, we used esiRNA to silence the expression of RhoV in A549 and PC9 cells. The transient silence effect was testified both at the mRNA level and protein level (**Figures 2A, B**). Using the CCK8 assay, we demonstrated that knockdown RhoV significantly reduced A549 and PC9 cell growth (**Figure 2C**), and the number of A549 and PC9 cells also had been decreased after RhoV deletion 72 h (**Figure 2D**). Meanwhile, the colony formation assay showed that LUAD cell growth activity markedly decreased after silence RhoV (**Figures 2E, F**).

Next, to further confirm the role of RhoV *in vivo*, we built the stable knockdown RhoV LUAD cell lines. Firstly, the knockdown efficiency has been manifested by western blotting (**Figure 2G**). Then, CCK8 data revealed that shRhoV cells grow slower than shScramble cells, and restoration of RhoV expression in RhoV knockdown cells rescued the inhibition of proliferation (**Figures 2H, I**). Besides, compared to A549 shScramble cells, we demonstrated that A549 shRhoV cells showed significantly smaller tumors, slower tumor growth speed, and lighter weight (**Figures 2J, K**). IF of Ki-67, one of the biomarkers of lung cancer progression, also exhibited lower expression in knockdown RhoV cells compared with knockdown negative control (**Figure 2L**). These data indicated that RhoV inhibited LUAD cell proliferation both *in vitro* and *in vivo*.

### Knockdown RhoV Suppressed LUAD Cells Migration and Invasion

**Figure 3:**
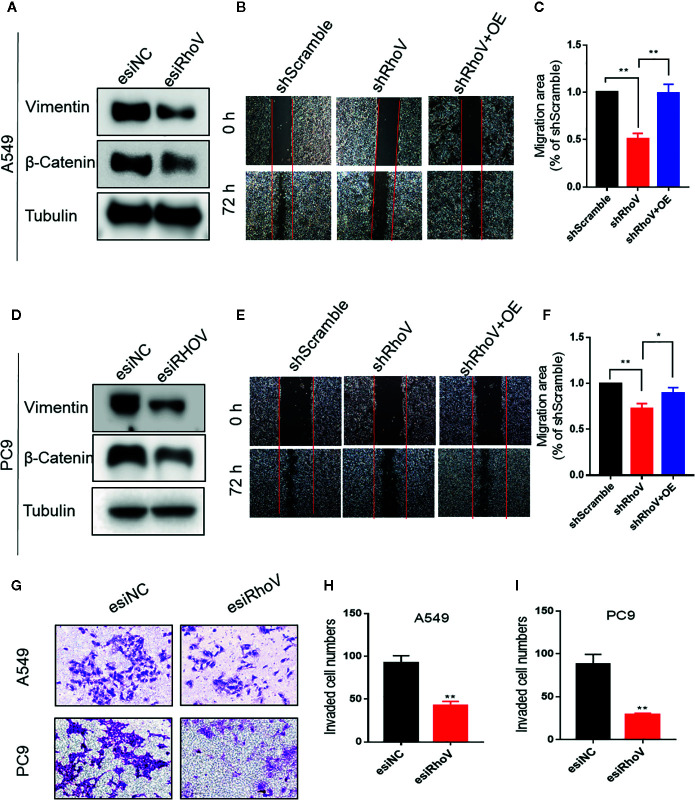
Knockdown RhoV suppressed A549 and PC9 cell migration and invasion. **(A, D)** Western blot analysis of Vimentin and β-Catenin expression after transfection esiRhoV for 48 h. Representative images of the scratch assay in A549 **(B, C)** and PC9 **(E, F)** cells and relative migration areas were measuring for statistics. **(G–I)** Representative images of transwell invasion assay in A549 and PC9 cells and the number of invaded cells were measuring for statistics. (Compared with match control, *p < 0.05, **p < 0.01).

Epithelial-mesenchymal transition (EMT) has been verified to drive morphogenesis and initiate tumor progression, mainly including cell migration and invasion phenotypes (15). Lots of Rho GTPase family members have been verified that could regulate cell migration and invasion (16, 17). The immunoblotting assay showed that Vimentin and β-Catenin, two biomarkers of EMT, had been decreased expression after silence RhoV (**Figures 3A, D**). Furthermore, based on the scratch assay, stable knockdown RhoV prohibited A549 and PC9 cell migration compared with the negative control group, and increasing RhoV in shRhoV cell could reversal this prohibition (**Figures 3B, C, E, F**). Moreover, RhoV deficiency suppressed A549 and PC9 cell invasion by transwell assay (**Figures 3G–I**).

### Downregulation RhoV Promoted Gefitinib Sensitivity in PC9 and PC9-GR Cells

**Figure 4:**
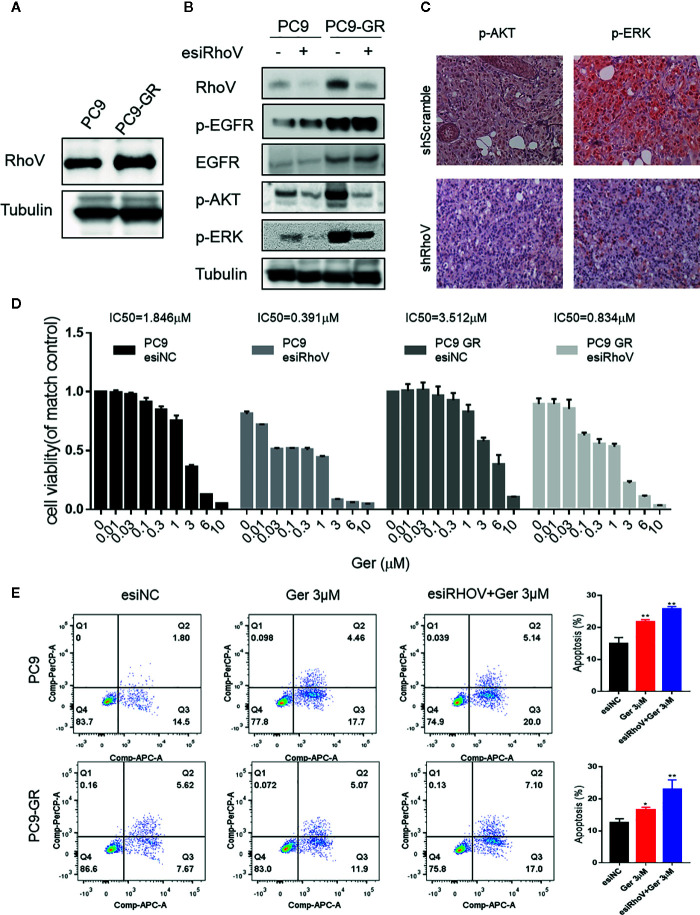
Silencing RhoV inhibited EGFR signaling pathway activation and promoted gefitinib sensitivity in PC9 and PC9-GR cells. **(A)** Western blot analysis of RhoV expression in PC9 and PC9-GR cell lines. **(B)** Western blot analysis of the mechanism of the EGFR signaling pathway after knockdown RhoV. **(C)** IHC analysis of p-AKT and p-ERK expressions in shScramble and shRhoV tumors. **(D)** CCK8 analysis of determining IC50 value against gefitinib of PC9 and PC9-GR cell with or without transfected esiRhoV. (Compared with match control, *p < 0.05, **p < 0.01.) **(E)** Flow cytometer analysis of gefitinib-induced PC9 and PC9-GR cells apoptosis percentage before or after transfected esiRhoV. (Compared with match control, *p < 0.05, **p < 0.01).

Gefitinib resistance is a serious problem for a significant percentage of lung cancer patients, especially for LUAD cases (18). The upregulation of RhoV in PC9-GR cells was confirmed by western blotting (**Figure 4A**). To confirm whether RhoV takes part in the EGFR pathway, we investigated the two classical downstream genes, AKT and ERK. As shown in **Figure 4B**, PC9-GR exhibited high phosphorylation of EGFR, AKT, and ERK in comparison to PC9. However, knockdown RhoV constrained these activities except EGFR phosphorylation. Meanwhile, the expression level of p-AKT and p-ERK were significantly decreasing in the shRhoV group compared with shScramble group *in vivo* (**Figure 4C**), which is consistent with *in vitro* results.

What is more, we detected the IC50 values against gefitinib and confirmed that PC9 was gefitinib-sensitive (IC50 = 1.846 μM), but PC9-GR was gefitinib-resistant (IC50 = 3.512 μM). Furthermore, silencing RhoV promoted gefitinib sensitivity both in PC9 (IC50 = 0.391 μM) and in PC9-GR (IC50 = 0.834 μM) cell lines (**Figure 4D**). Moreover, down-expression RhoV aggravated gefitinib-induced PC9 and PC9-GR apoptosis by flow cytometer assay (**Figure 4E**). These findings indicated that RhoV regulated the EGFR pathway to administrate the effect of gefitinib sensitivity in LUAD.

## Discussion

Lung adenocarcinoma (LUAD) is one of the serious cancer types worldwide, and the mechanism is still unrecognized. Continuous attentions concentrate on tumor suppressor genes, which participated in the process of lung cancer. Their core role and basic contribution to tumor cell behavior have become more and more clear mechanisms, as well as there still are major challenges to the treatment of LUAD. Nowadays, accumulating researches indicate that the GTP-binding proteins dependent cell signaling pathway is important for malignant carcinoma development migration and the motility process function in LUAD. Rho GTPases family, as key regulators of cytoskeletal rearrangement and a subfamily of the Ras superfamily, has raised considerable concern. Increasing evidence showed that Rho GTPases, like RhoA (19), cdc42 (20), and Rac1 (21), are involved in LUAD progression and metastasis.

In the present study, we focused on RhoV, one atypical Rho GTPase, which functions are insufficiently characterized. Using bioinformatics analysis, we gathered the 525 DEGs in LUAD with three analysis methods. We investigated that RhoV showed significantly increased expression in LUAD cases compared with normal cases. Besides, RhoV was enriched in glyoxylate and dicarboxylate metabolism; the reason might be it could regulate GTP activation and cell adhesion (**Figures S1E, F**). Moreover, the up-expression of RhoV leads to poor overall survival and progression-free interval in LUAD. However, the lung squamous cell carcinoma (LUSC) overall survival time is the opposite for LUAD. This seems that RhoV in different lung cancer types displayed different functions (data not shown), which should be further researched. Further data showed that the expression of RhoV in patients with LUAD was overexpressed compared with match normal. Meanwhile, RhoV was expressed more prevailingly in LUAD cell lines compared to BEAS-2B cells. This is consistent with Mikhail et al.’s report in 2013 (9). According to these initial data, RhoV might be a possible therapeutic target and a negative prognostic factor of LUAD.

Furthermore, knockdown RhoV, by esiRNA or plasmids, was exhibited great inhibition of LUAD cell proliferation *in vitro*, meanwhile inhibited the formation of tumor growth *in vivo*. The important tumor proliferation biomarker, Ki-67 (22), was degraded by RhoV depletion, such as this phenomenon was by cell viability decreases. Through a series of *in vitro* and *in vivo* experiments, we testified the correlation of RhoV and lung cancer cell progression or tumorigenesis. Regarding RhoV function in cancer cells, RhoV has been verified that alters cell shape and cell adhesion (23). In this study, silencing RhoV inhibited the migration and invasion in LUAD cells. Meanwhile, RhoV depletion exhibited the two EMT biomarkers, Vimentin and β-Catenin, expressions. Notably, after knockdown RhoV, we found that E-cadherin also reduced expression (**Figure S2**). One report showed that RhoV maintains E-cadherin at adherens junctions during zebrafish epiboly (24), which coincides with our study. E-cadherin has been confirmed that is one of the great effective suppressors for cancer EMT. For our discovery, even deficiency RhoV induced E-cadherin decreasing, A549, and PC9 migration and invasion levels still weaken compared with the control group. These data indicated that RhoV might switch on or switch off other genes than to control EMT progress, not only in the E-cadherin pathway. This question should be the focus of further research.

EGFR is a key receptor protein that is present on the surface of both normal cells and cancer cells. More and more studies showed that EGFR is a valuable therapeutic target for many cancers, especially in LUAD (25). Gefitinib is one of the best treatments with advanced NSCLC thought inhibited EGFR activation (26), which has been confirmed for more than 10 years in the clinic trial assay. However, an increasing number of lung cancer patients exhibited resistance to gefitinib, which leads to worse survival and difficult treatment. Still now, the resistance of the gefitinib mechanism is confusing. Gefitinib-resistant (GR) patients manifest many pathways abnormal activation, including MET/HER2 amplification, activation of the RAS-mitogen-activated protein kinase (MAPK)-ERK, or RAS-phosphatidylinositol 3-kinase (PI3K)-AKT pathways (27–29). In this study, we found that PC9-GR cells showed a high expression of RhoV, which means RhoV might associate with GR circumstance. To delve further into the potential inhibition of RhoV in GR, we checked IC50 of gefitinib *in vitro* and investigated that expression of RhoV decreased gefitinib sensitivity both in PC9 and PC9-GR cells. Furthermore, RhoV knockdown also prevented EGFR-downstream activation, both phosphorylation of ERK and AKT, as well as promoted gefitinib-induced cell death. However, EGFR phosphorylation seemed to not significantly change after RhoV down-expression.

## Conclusion

Overall, the current study investigated RhoV as an important and new participant in LUAD. Moreover, RhoV is involved in tumor cell proliferation and EMT both *in vivo* and *in vitro*. Additionally, RhoV regulated gefitinib sensitivity in GR cells. Remarkably, RhoV knockdown also altered some of the suppressor’s expression in tumor progression. This suggested that RhoV might have some feedback on protective effects, which is needed to do more research and validate the mechanism pathway in the future.

## Data Availability Statement

The original contributions presented in the study are included in the article/**Supplementary Material**. Further inquiries can be directed to the corresponding authors.

## Ethics Statement

The studies involving human participants were reviewed and approved by Nanjing Medical University. The patients/participants provided their written informed consent to participate in this study. The animal study was reviewed and approved by Nanjing Medical University.

## Author Contributions

HC and HXi conceived the study and performed experiments. RX, LJ, YZ, GZ, YW, and YJZ helped the sample collection and investigation. HXu, CY, and WP support experiments and data analyses. HC and HXi drafted the manuscript and revision. All authors contributed to the article and approved the submitted version.

## Funding

This study was supported grants from The Recruitment Program of Overseas High-Level Young Talents, “Innovative and Entrepreneurial Team” (No.(2018) 2015), Science and Technology Grant (BE2019758) and the Six Talent Peaks Project (TD-SWYY-007) of Jiangsu Province and High-Level Talents Program of Nanjing Medical University; Funding of Postdoctoral; Funding of Nanjing Drum Tower Hospital Affiliated to Medical School of Nanjing University, The Medical Interdisciplinary Research Funding of Henan University (NO. CJ1205A0240011). Postgraduate Research & Practice Innovation Program of Jiangsu Province (SJKY19_1327 to HC), China Scholarship Council (201908320572 to HC), and Nanjing Medical University Scholarship (C124 to HC).

## Conflict of Interest

The authors declare that the research was conducted in the absence of any commercial or financial relationships that could be construed as a potential conflict of interest.
