# Supplementary material for: Overexpression of RhoV Promotes the Progression and EGFR-TKI Resistance of Lung Adenocarcinoma
Source: Front Oncol. 2021 Mar 9;11:619013. doi: 10.3389/fonc.2021.619013 (PMC7986718; doi:10.3389/fonc.2021.619013)
Supplement: Supplementary file 1 [file DataSheet_1.docx]

**Overexpression of RhoV promotes the progression and EGFR-TKI resistance of lung adenocarcinoma**


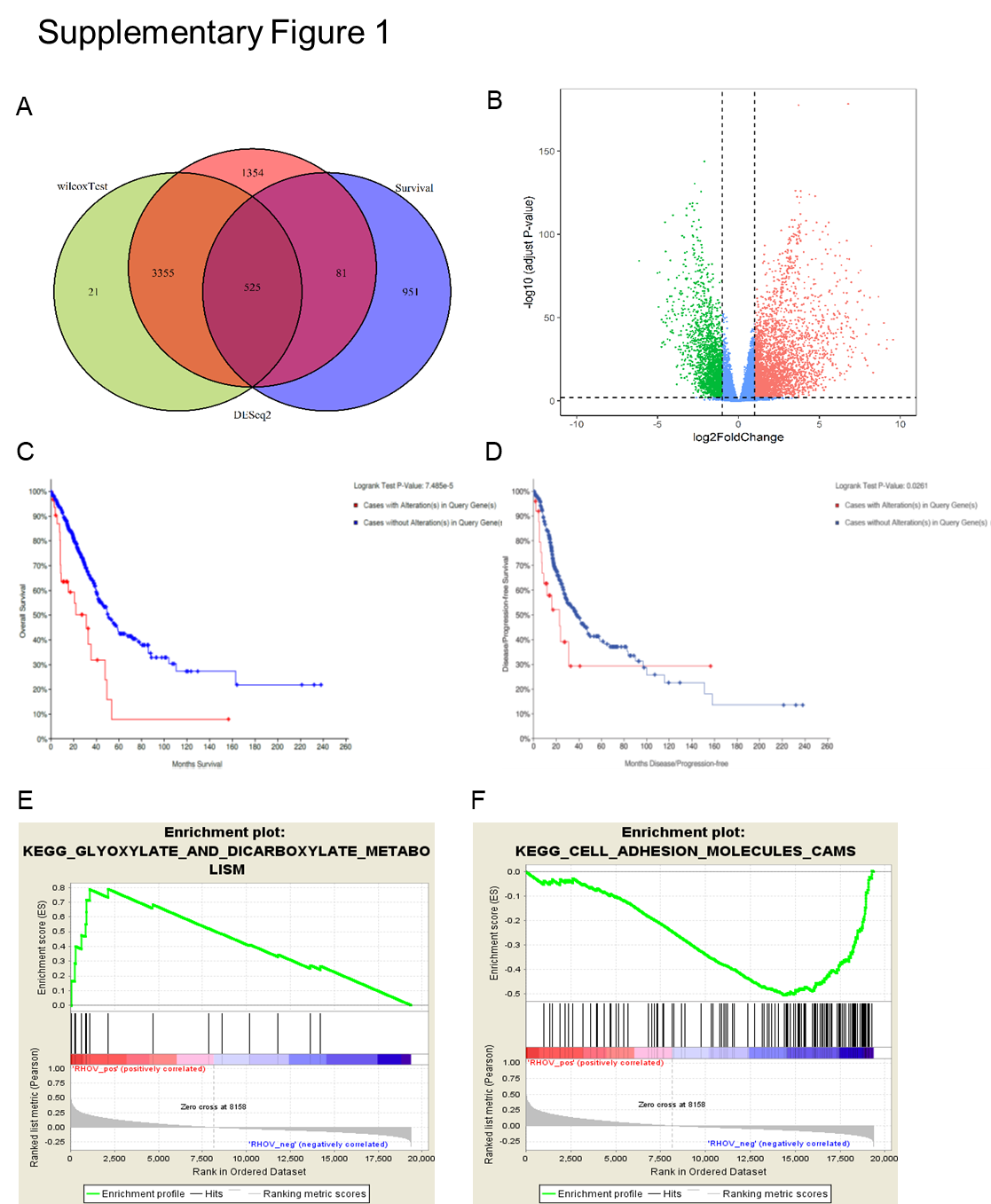
**Supplementary Figure 1. Bioinformatics analysis of RhoV.** (A) WilcoxTes, edgeR and survival analysis of 481 LUAD tumor tissues and 59 normal lung tissues data which were downloaded from TCGA. Then, Venn diagram collected 525 cross differential genes. (B) Visual hierarchical cluster analysis of the differential genes in LUAD via Volcano plot. (C-D) Kaplan–Meier analysis of overall survival and disease-free survival about different RhoV mRNA expression in LUAD at months thought cBioPortal. (E-F) GSEA analysis of RhoV mainly increased glyoxylate and dicarboxylate metabolism and cell adhesion pathways.


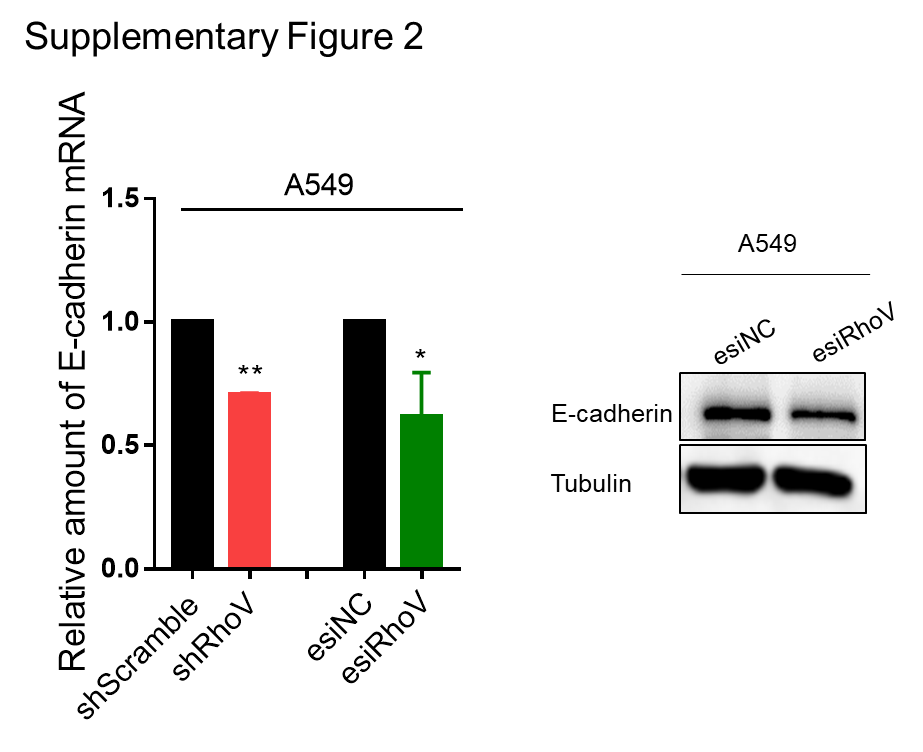
**Supplementary Figure 2. Knockdown RhoV inhibited E-cadherin expression.**

(A) qRT-PCR and western blot analysis of knockdown RhoV decreased the mRNA and protein expression of E-cadherin in A549 cells. (Compared with match control, p*<0.05, p**<0.01)
